# Complex hierarchical microstructures of Cambrian mollusk *Pelagiella*: insight into early biomineralization and evolution

**DOI:** 10.1038/s41598-017-02235-9

**Published:** 2017-05-16

**Authors:** Luoyang Li, Xingliang Zhang, Hao Yun, Guoxiang Li

**Affiliations:** 1 Shaanxi Key Laboratory of Early Life and Environments, State Key Laboratory of Continental Dynamics, and Department of Geology, Northwest University, Xi’an, 710069 P.R. China; 2 SKLPS, Nanjing Institute of Geology and Palaeontology, Chinese Academy of Sciences, Nanjing, 210008 P.R. China

## Abstract

Shell microstructure and mineralogy of *Pelagiella madianensis* Zhou & Xiao, 1984, a globally distributed Cambrian micromollusk, are investigated based on abundant and extraordinarily well-preserved specimens from Xinji Formation, Longxian, Shaanxi, North China. Five types of aragonitic microstructures have been recognized. The lamello-fibrillar microstructure, previously known from *Pelagiella*, constructs the outermost shell layer, while the remaining four types are reported here for the first time in this genus. They include fibrous foliated, foliated aragonite, crossed foliated lamellar and isolated tablets. The animal constructs these five types of microstructures to build its shell in a complex hierarchical pattern with four orders: crystallite columns, laths, folia and lamellae. These findings demonstrate that the capability of building complex shell microstructures had already evolved by the Cambrian explosion. In addition, this work shows that early aragonitic shells were constructed with fibers, laths, folia and isolated tablets, indicating increased controls over biomineralization by the animal.

## Introduction

Molluscan shells (bivalves, gastropods and monoplacophorans) initially evolved during the earliest Cambrian (beginning at 541 Ma)^1, 2^ to protect the soft tissues from abiotic and biotic stress^3^. They were diverse in the Cambrian, both in morphology and microstructure. At least 160 genera have been described from the lower Cambrian (Terreneuvian plus Cambrian Series 2)^4^ and the number is still increasing^5, 6^. Much attention has previously been paid to the taxonomy, phylogeny, and biostratigraphy of early molluscan shells^7, 8^. However, micro-nano scale microstructure and the mineralogy of shells have been poorly determined. Since the pioneering work of Runnegar^9^, microstructures of Cambrian molluscan shells were known from phosphatic internal moulds^10^. Many types of microstructures found in modern molluscan shells (*e*.*g*. foliated calcite, foliated aragonite, fibrous and prismatic forms) can be traced back to the Cambrian^11^, indicating a rapid diversification of the lineage. Nevertheless, complex shell microstructures such as crossed lamellar and nacre have not yet been discovered in the Cambrian. In addition, the lack of detailed sub-units of already known microstructures also limits understanding of biomineralization of early molluscan shells and restricts comparisons with modern types.

One of the difficulties in determining shell microstructures of Cambrian mollusks is distinguishing and reconstructing a variety of thin layers, which appear to be preserved primarily on the surface of phosphatic internal moulds after acid-etching^12, 13^. The most frequently used model is the two-layered structural sequence, as Cambrian molluscan shells consisted of outer prismatic and inner lamellar layers^10, 11^. However, investigations into shell microstructures of living mollusks have shown that some limpet shells have up to five layers with distinctive microstructures observed in transverse section through the thickness of the shells^14, 15^.

*Pelagiella* is a globally distributed Cambrian micromollusk that has a turbospiral dextrally coiled shell and a sub-triangular aperture. Biological affinities of *Pelagiella* are still uncertain mainly because of the lack of anatomical evidence for torsion, and it has been interpreted as a gastropod, a paragastropod or a helcionellid^16^. Shell microstructure is lamello-fibrillar, which consists of fine parallel fibers arranged into a mosaic pattern (radially and comarginally oriented aragonitic crystals)^11, 17^. Additionally, flattened, bladed crystallites aligned with the growth direction were also reported from the inner shell layer of *P*. *deltoides* ^9^ and *P*. *subangulata* ^3, 18^. This demonstrates that their shells consist of complex microstructures, including various types of crystallites and arrangements.

Here, we document additional information on shell microstructures of *P*. *madianensis* from the Xinji Formation (equivalent to uppermost Cambrian Stage 3) of the lowermost Cambrian of the North China block^19, 20^, including detailed architecture through the shell. These data will be put into a general evolutionary context of early molluscan biomineralization.

## Results

### Mode of Preservation

The replacement of original skeletons by calcium phosphate (“phosphatization”) is of great significance in the preservation of Cambrian Small Shelly Fossils. Phosphate can produce a perfect pseudomorph and replicate very fine features of other materials, such as delicate microstructures of shells and muscular filaments of soft tissues^21, 22^. However, preservation of mollusks is strongly dependent on rock type, as well as on the method of fossil extraction^23^. Specimens are generally preserved as phosphatic internal moulds, as the calcareous shells are dissolved during the acid leaching extraction process (see Supplementary Fig. S1). Consequently, preservation of shell microstructures, especially on micro-nano scales, is extremely scarce in early molluscan shells as well as other skeletal fossils.

**Figure 1:**
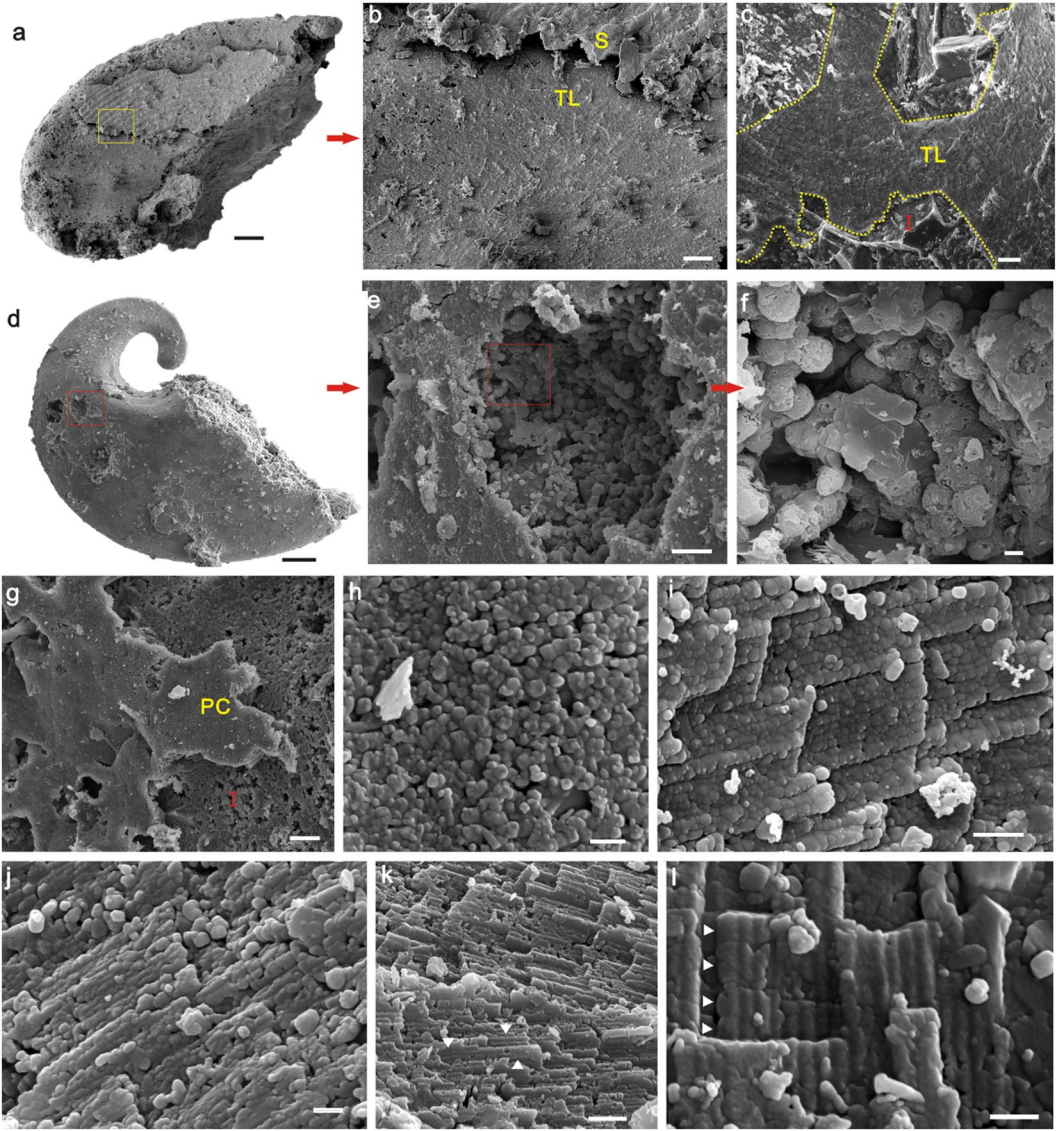
Mode of Preservation. (**a**,**b**) LC063267, acid-etched shell residue (S) and Thin-layer (TL) of foliated aragonite, (**c**) LC063163, Thin layer (TL) of foliated aragonite marked by yellow dotted line, (**d**–**f**) LC063046, shows internal mould (I) and microbial induced spheroids, (**g**) LC063026, acid-etched shell residue and internal mould, (**h**) LC063043, amplification of outer shell surface shows the diagenetic apatite, (**i**) LC063522 and (**j**) LC063519, over-phosphatized shell remains the pseudomorph of lath-like crystals, but composed of diagenetic apatite, (**k**) LC063097 and (**l**) LC063055, crystals grow in typical prismatic form (white arrowhead). Scale bars of (**a**,**d**) are 100 μm; (**b**)20 μm; (**c**,**e**,**g**)10 μm; (**f**,**i**,**k**)1 μm; (**h**,**j**,**l**)500 nm.

Most specimens of *Pelagiella* from the Xinji Formation that are preserved as internal moulds are composed of phosphatic spheroids (2–5 μm in diameter) (Fig. 1d–f), likely to have been formed by microbial activity. Lamellar microstructure of the shell is readily discernable and distinguishable from the internal moulds by characteristic pseudomorphic laths of aragonite, which grow consistently in a radial direction and have complex hierarchical orders (Fig. 1a–c). When magnified, some specimens show the crystallite columns stacked parallel to each other inside the lath (Fig. 1k).

The high resolution SEM images reveal a variety of nano-crystals of calcium phosphate (mainly apatite) both from phosphate coatings and shell residues. In most cases, these nanograins are 150 nm in diameter (Fig. 1i), and are irregularly arranged inside the laths, and resemble the aragonitic nanogranules of original molluscan shells (see Supplementary Fig. S2). However, nanograins are also common in the phosphate coatings (Fig. 1g,h). In these cases, each nanograin seems to be composed of smaller structures (Fig. 1j–h). Apatite crystals typically grow in a prismatic form, 50–200 nm in diameter and 350–500 nm in length (Fig. 1k,l). Consequently, it is difficult to determine whether original aragonitic nanogranules are retained during the process of phosphatization. In contrast, the apatite nanograins are very likely to be formed during diagenesis.

### Shell morphology

**Figure 2:**
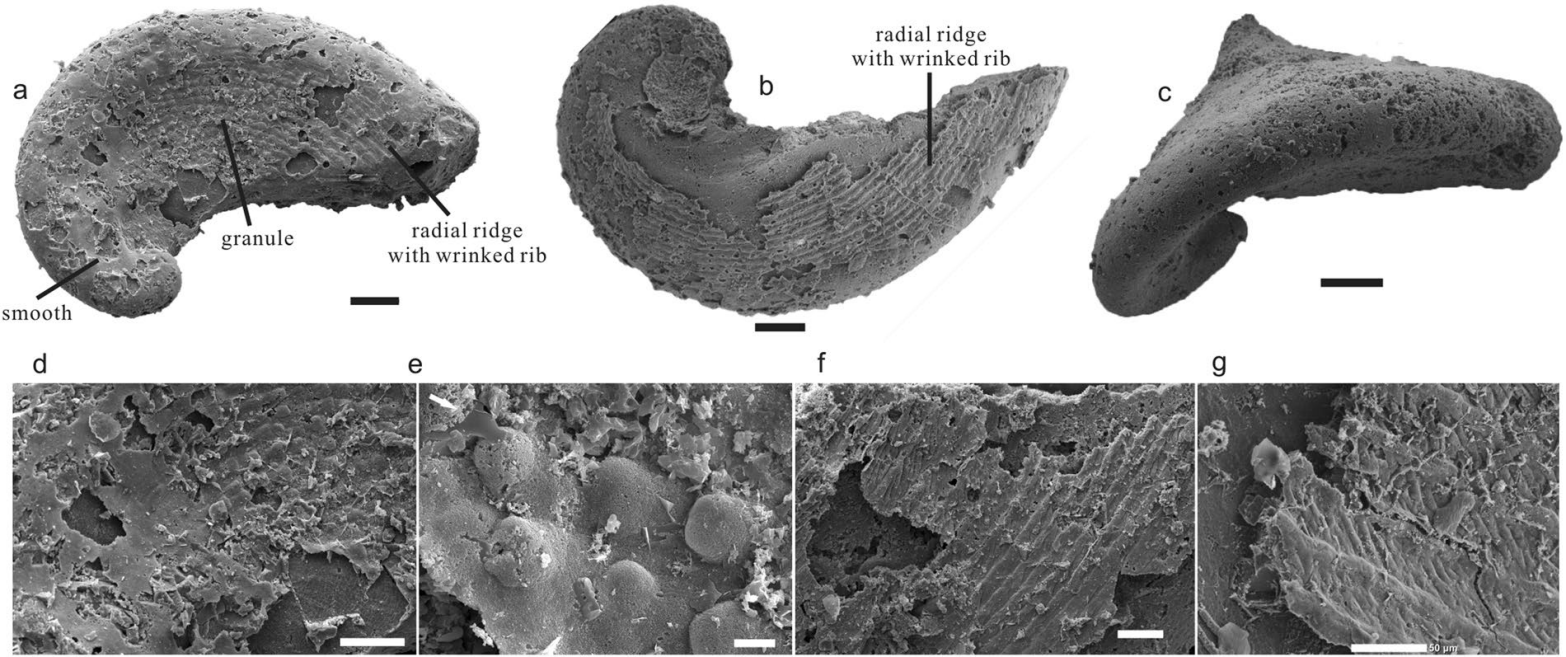
Morphology and ornaments of *P*. *madianensis*. (**a**) LC063505 (**b**) lc060159 (**c**) lc060326 shows the turbospiral, dextrally coiled shell with an open umbilicus and elongated aperture, (**d**) Amplification of (**a**), the smooth surface of the front part, (**e**) LC063043, shows isolated knobs aligned in radial direction, arrow indicates radial direction, (**f**) LC063521 and (**g**) lc060138, crossed ridges with wrinkled ribs of the last whorl. Scale bars of (**a**–**c**) are 100 μm; (**e**) 5 μm; (**d**,**f**,**g**) 50 μm.

*Pelagiella madianensis* has a turbospiral, dextrally coiled shell with an open umbilicus and an elongated aperture separated from the well-rounded dorsum by the depressed lateral shell (Fig. 2a,b). As a result, the shell is funnel-like in plane view and the spire is slightly submerged (Fig. 2c). Shell exterior can be finely ornamented by three distinctive sculptures with the development of the shell: smooth (Fig. 2d), knobs (Fig. 2e) and radial ridges with wrinkled ribs (Fig. 2f,g). The cross-section of shell from spire to aperture is elongated and rectangular to sub-triangular in outline.

### Shell microstructures

**Figure 3:**
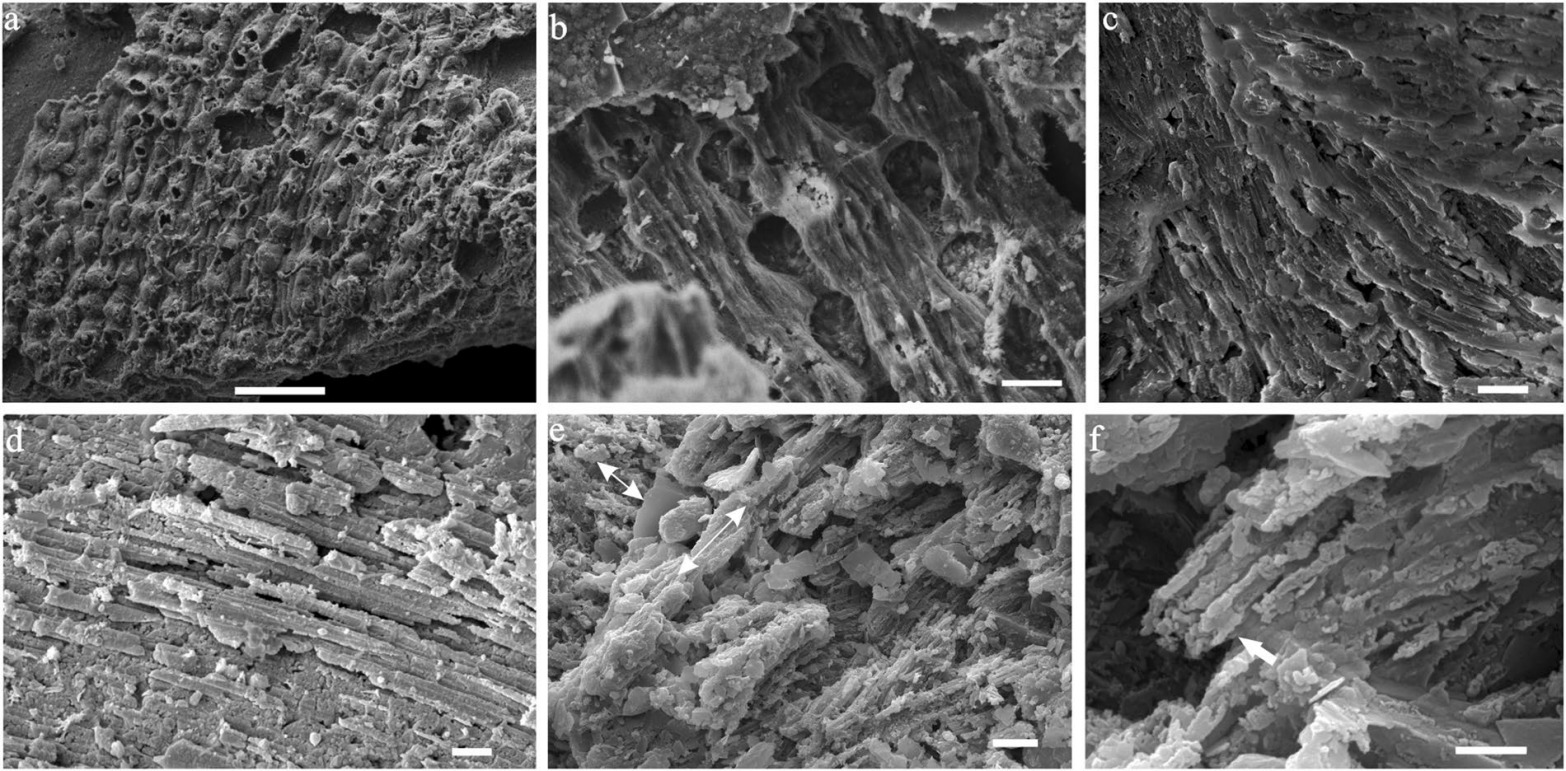
Lamello-fibrillar microstructure of *P*. *madianensis*. (**a**) lc060328 and (**b**) lc060145, exterior surface and interior surface of the shell with ornament of radial knobs with wrinkled ribs, (**c**) LC063157 and (**d**) LC063266, fibers of lamello-fibrillar are mainly aligned in radial direction, (**e**,**f**) LC063039 Mosaic pattern of lamello-fibrillae with fibers respectively aligned in radial and comarginal direction (double-headed arrows), white arrow in (**f**) indicates recrystallized fiber. Scale bars are (**a**) 10 μm; (**b**–**e**) 5 μm; (**f**) 1 μm.

The lamello-fibrillar microstructure occurs throughout the shell. It is a thin layer that is composed of continuous fibers, or bundles of fibers parallel to the shell surface, aligned in a radial direction (Fig. 3a–d). These fibers may be branched or crossed, which corresponds to the wrinkled rib sculpture (Fig. 3c). Bundles of fibers may be more or less arranged in the comarginal direction (Fig. 3e,f).

**Figure 4:**
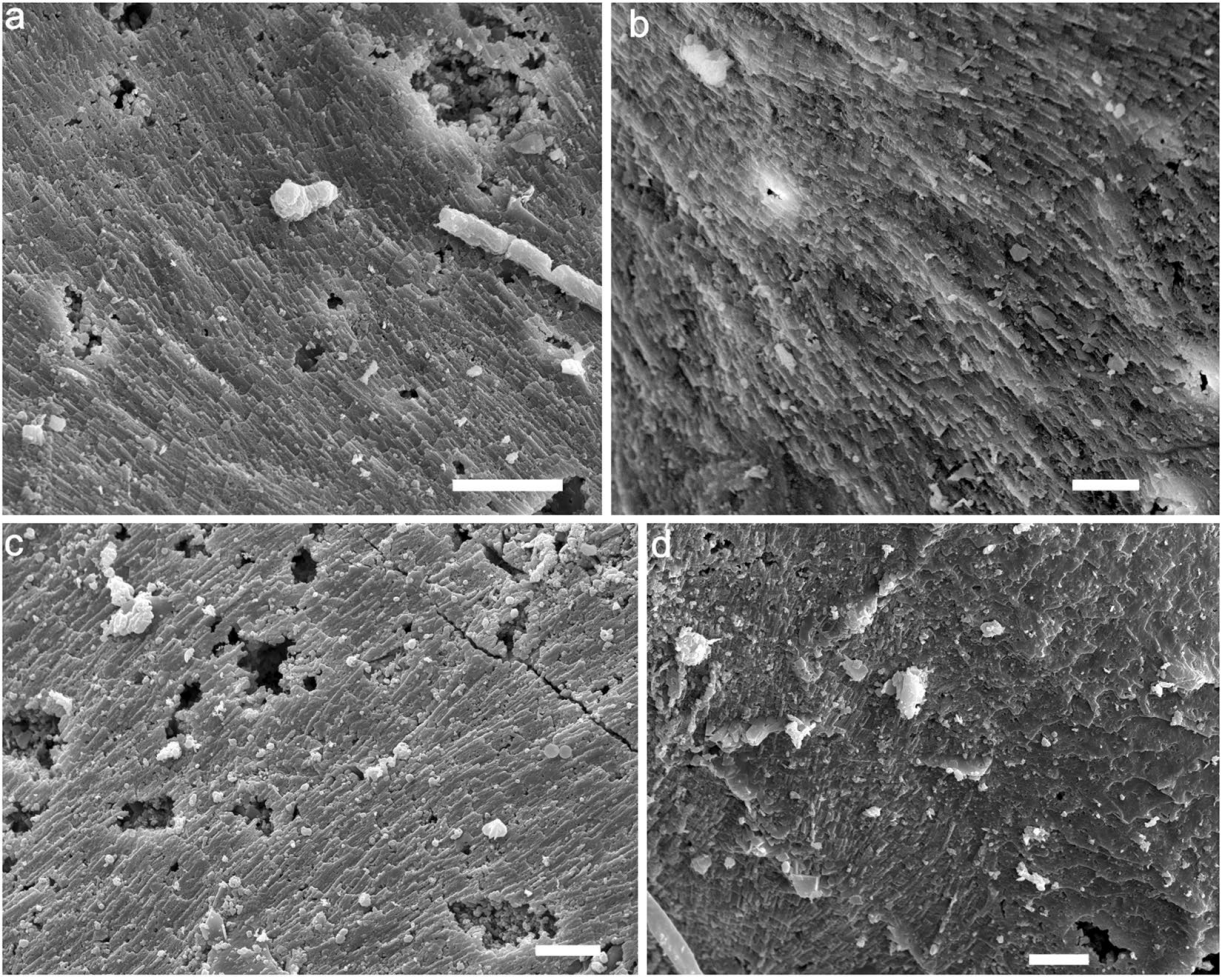
Fibrous foliated microstructure of *P*. *madianensis*. (**a**) LC063097 (**b**) LC063172 (**c**) LC063519, the fibrous appearance and acicular laths arranged in radial direction, (**d**) LC063042, distinctive fibrous appearance of fibrous foliated and consistent pattern of foliated aragonite. Scale bars are (**a**–**d**) 5 μm.

Underlying the outermost shell layer, especially within the umbilical wall, crystals are elongated, narrow, acicular laths with straight fronts, unlike the fibers of lamello-fibrillae or the wide laths of foliated aragonite (Fig. 4a,b). These crystals have a width of about 1–2 μm and are aligned into fibrous pattern in gross appearance (Fig. 4c,d). Hence this type of microstructure is described as “fibrous foliated”.

**Figure 5:**
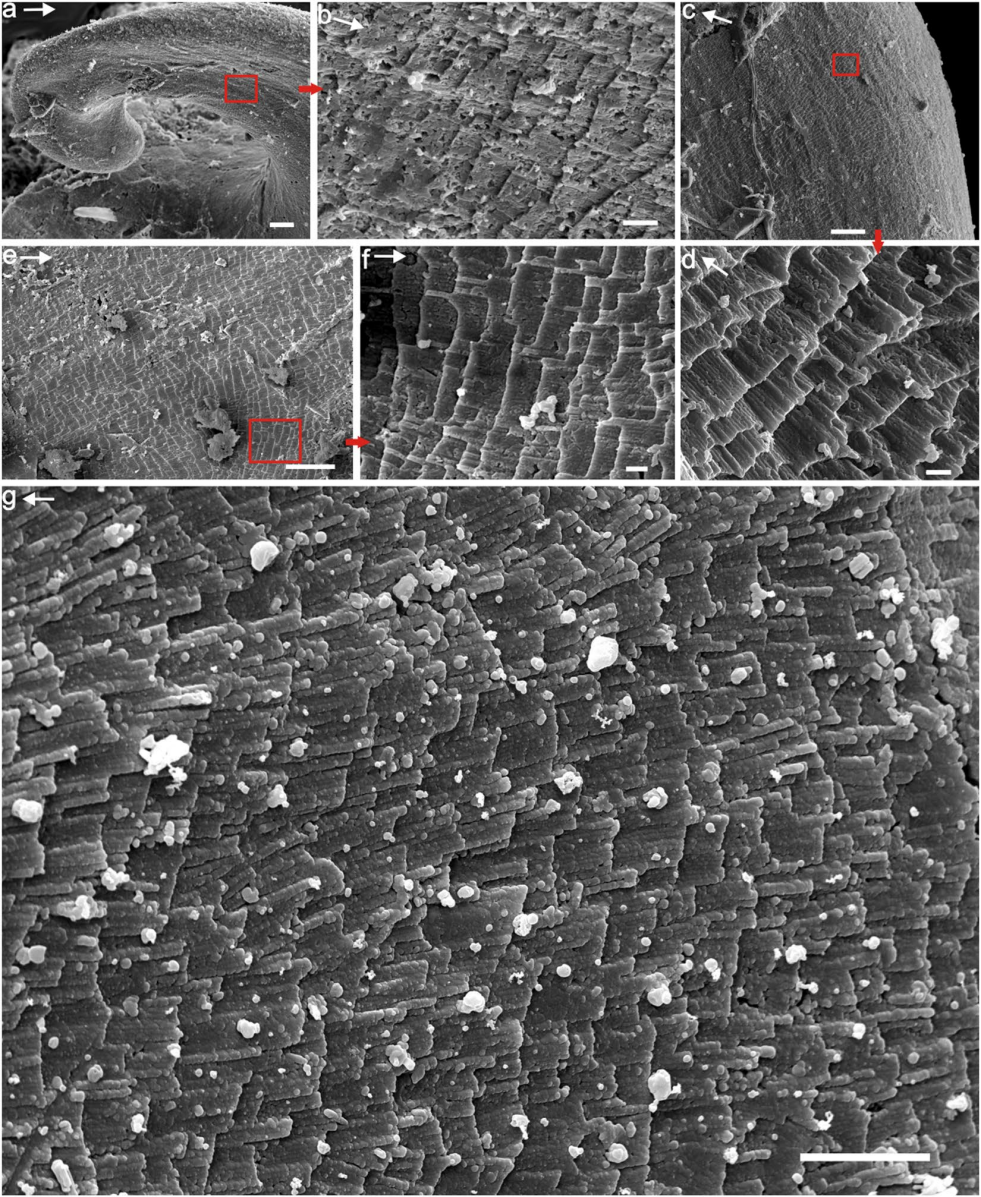
Foliated aragonite microstructure of *P*. *madianensis*. (**a**,**b**) LC063024, shows the regular pattern of foliated aragonite in radial direction, (**c**,**d**) LC060011, the second-order folia with straight front, (**e**,**f**) LC063267, stepwise second-order folia aligned in radial direction, (**g**) LC063522, shows the third-order laths and fourth-order crystallite columns arranged in radial direction. Scale bars are (**a**,**c**) 20 μm; (**b**,**e**) 10 μm; (**d**,**f**) 1 μm (**g**) 5 μm. White arrows indicate the growth (radial) direction.

The foliated aragonite microstructure is widely distributed, but mainly preserved in the internal layer of the early-secreted shell. Its crystallites are always arranged in the radial direction, and three to four hierarchical orders can be identified. The second-order sheets (folia) show a stepwise pattern, margined with straight front (Fig. 5d–f). Third-order laths are parallel to each other inside the folia with width ranging from 2 to 6 μm and 50–200 nm in thickness (Figs 1k,l and 5g). Upon closer examination, numerous fourth-order crystallite columns (50–200 nm in diameter) can also be identified from each lath, which are also parallel to each other (Fig. 5d,g). The foliated aragonite shows a regular pattern unlike the fibrous foliated microstructure (Fig. 5a–c).

**Figure 6:**
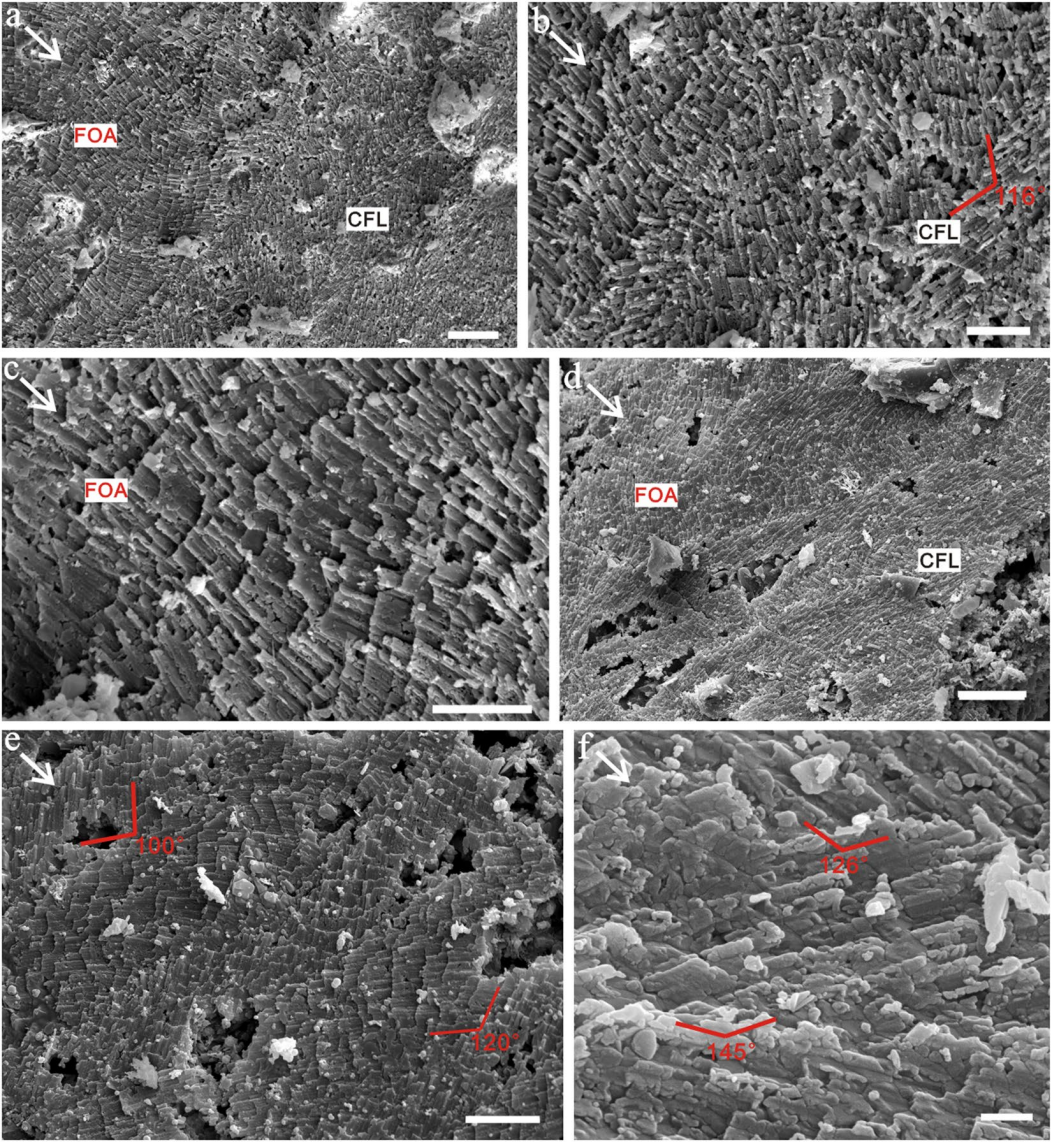
Initial layer of crossed foliated lamellar microstructure. (**a**) LC063303, the transition from foliated aragonite (FOA) to crossed foliated lamellar microstructure (CFL), (**b**) Amplification of (**a**), shows the initial layer of crossed lamellar microstructure, (**c**) Amplification of (**a**), shows the foliated aragonite microstructure, (**d**) LC063519, The orientation of laths rotates from radial to comarginal direction from the FOA to CFL, (**e**) LC063047 and (**f**) LC063097, The angle (from plain view) between adjacent first-order lamellae increases from almost 90 to 145°. Scale bars are (**a**,**d**) 10 μm; (**b**,**c**,**e**) 5 μm; (**f**) 1 μm. White arrows indicate the growth (radial) direction.

**Figure 7:**
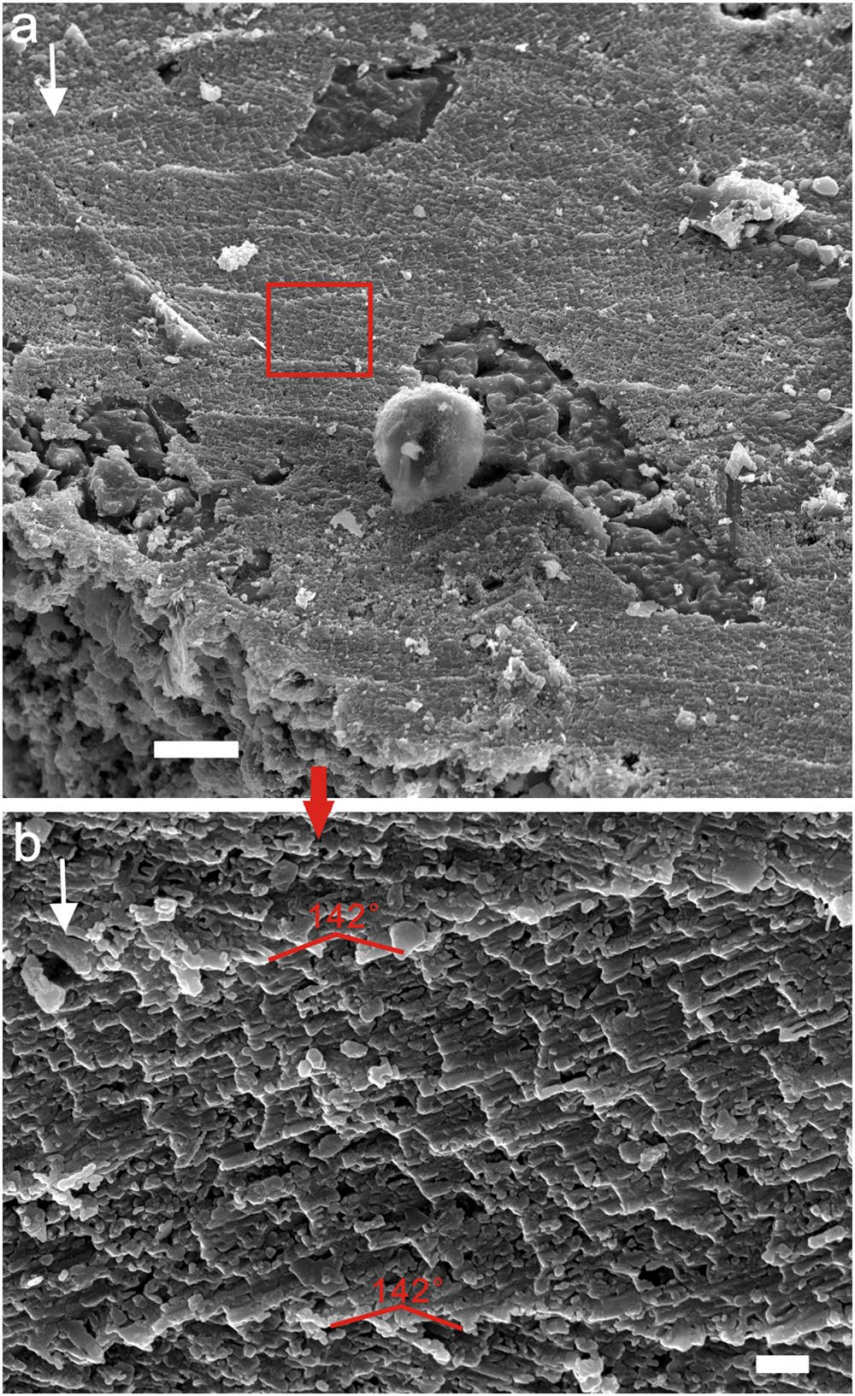
Mature crossed foliated lamellar microstructure. (**a**) LC063006, the first-order lamellae are secreted comarginally; folia and laths parallel growth surface, (**b**) Amplified SEM image shows the basic units of crystallite columns and laths arranged oppositely to adjacent first-order lamellae. Scale bar are (**a**) 10 μm; (**b**) 1 μm. White arrows indicate the growth (radial) direction.

Crossed foliated lamellar microstructure (CFL) is observed close to the aperture of larger specimens and shows a fourth-order hierarchical pattern from lamellae to crystallite columns. When the CFL is laid down below the foliated aragonite layer (in the direction of radial shell accretion), the orientation of laths rotate from a radial to a comarginal direction (Fig. 6a–c). Its initial layer has a width of about 2–5 μm and is composed of acicular crystallites (Fig. 6d–f). The first-order lamellae are comarginally arranged. The width of the first-order lamellae is up to 10 μm (Fig. 7a). The second-order folia and third-order laths grow in opposite directions with the angle between them (from plain view) increasing from almost 90° to 145° (Fig. 7b).

**Figure 8:**
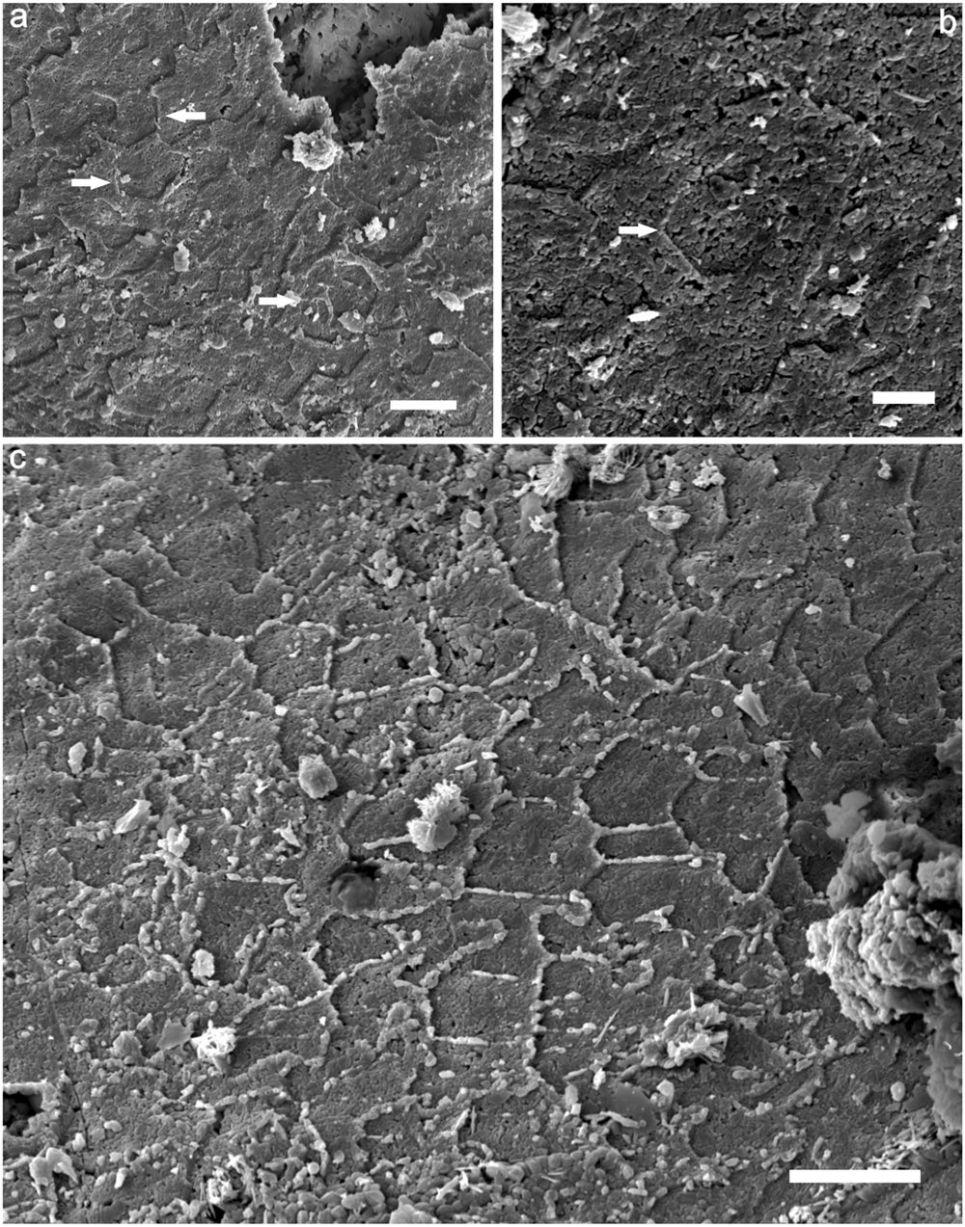
Isolated tablets embedded in foliated aragonite microstructure. (**a**) LC06–036 (**b**) LC06-048, SEM images show the isolated hexagonal tablet with a 120 interfacial angle, (**c**) LC06-085, Transition from lath or folia with straight fronts through pseudo-hexagonal fronts to hexagon outline of the crystal. Scales bars are (**a**–**c**) 5 μm.

Isolated tablets (5–10 μm in diameter) co-occur with foliated aragonite microstructure in the early-secreted shell, and are mainly restricted within the innermost shell layer (Fig. 8c). However, only the outline of the crystallites (tablets and folia) is preserved, and they do not display any detailed sub-units. The third-order laths of foliated aragonite have a pseudo-hexagonal outline with a 120° interfacial angle in contrast with the straight front of the second-order folia where space for growth is generally unlimited (Fig. 8a,b). In addition, imprints of completed hexagonal crystals can be easily identified, and the transitions of growing fronts from straight to hexagonal are gradual.

## Discussion

**Figure 9:**
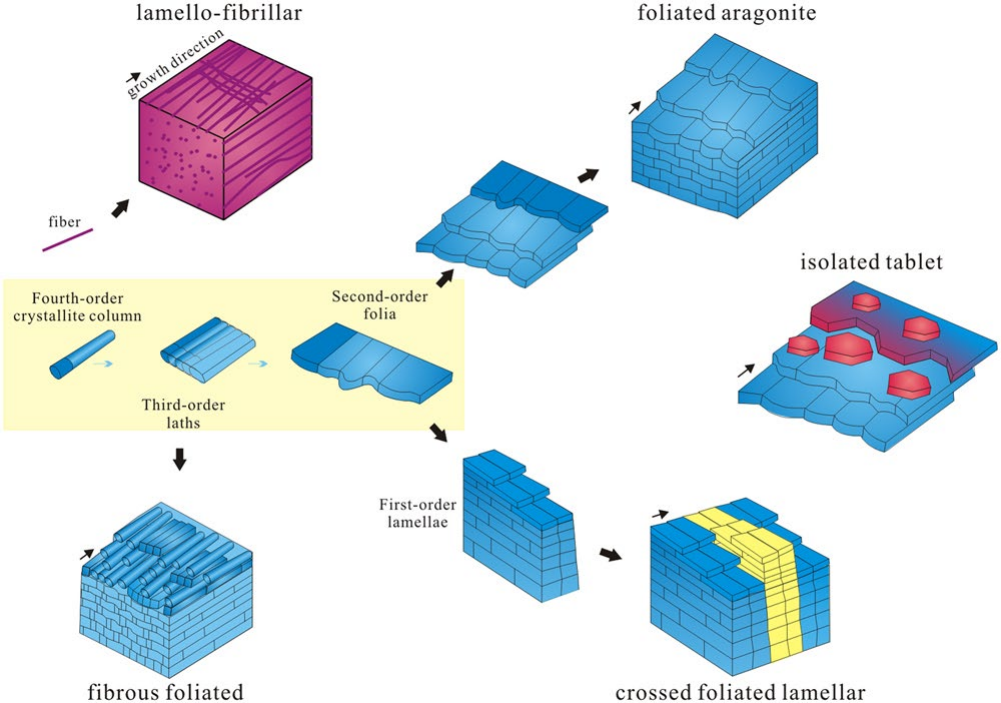
Reconstruction of shell microstructures. Basic units with four hierarchical orders: lamellae, folia, laths and crystallite columns construct five types of microstructures: lamello-fibrillar, fibrous foliated, foliated aragonite, crossed foliated lamellar and isolated tablets.

Four hierarchical orders of shell construction (*i*.*e*. from crystallite columns to lamellae) in *P*. *madianensis* are revealed by a variety of microarchitectures and crystal orientations. In addition, five types of microstructures are also recognized in phosphatic internal moulds and phosphate-replaced shells, *i*.*e*. lamello-fibrillar, fibrous foliated, foliated aragonite, crossed foliated lamellar and isolated tablets. This demonstrates that the capability of building complex shell microstructures had already evolved by the period of the Cambrian explosion (Fig. 9).

The precise position of each type of microstructure is difficult to determine because boundaries or discontinuities between adjacent layers are gradational, and are particularly hard to observe on the horizontal surface of the shell. However, in selected specimens, these five types of microstructures may be preserved within specific parts of the shell (Figs 2d, 3a, 4d, 6a,d and 8c). This demonstrates that multiple layers with distinctive lamellar microstructures occur within these shells, in contrast to the classical two-layered model of this Cambrian micromolluscan species.

The lamello-fibrillar microstructure previously observed in *Pelagiella subangulata* Tate, 1982 constructs the outermost shell layer of *P*. *madianensis* (Fig. 2b). However, the mosaic pattern of this type of microstructure is not consistently observed in all specimens. Evidence from *P*. *subangulata* and *P*. *deltoides* shows similar crystals mainly arranged in the same radial direction^9, 18^. In addition, comarginal grooves on internal moulds of *P*. *subangulata* (see Bengtson *et al*. 1990, Fig. 169. C) seem to be composed of acicular laths in a crossed foliated lamellar pattern, rather than fibers of lamello-fibrillae. Lamello-fibrillar microstructure is observed in *P*. *madianensis* based on limited data of comarginal fibers, however additional investigation is required, particularly because detailed subunits of the fibers are unclear as a result of diagenesis. It is possible that the transition during growth from radially-arranged fibers of lamello-fibrillae to wide laths of foliated aragonite is gradual.

Narrow, elongated crystallites reinterpreted as acicular laths of fibrous foliated microstructure were previously documented from the inner shell layer of *P*. *deltoides*, but they were initially interpreted as fibers of lamello-fibrillae^9^. Actually, the acicular laths represent an intermediate form between fibers to laths of aragonite. As a result, the fibrous foliated microstructure is determined as a transitional stage from lamello-fibrillar to foliated aragonite in *P*. *madianensis*. This microstructure shares close similarities with the regular or irregular fibrous foliated microstructures of recent Patellogastropoda^14^ as both have bladed units and a fibrous appearance. However, the straight front of acicular laths in the fibrous foliated microstructures documented herein is a texture associated with aragonite rather than the calcitic arrow-like laths in Patellogastropoda.

Foliated aragonite microstructure was initially described from extant monoplacophorans^24, 25^, but recent work revealed that it also occurred in the Cambrian bivalves *Pojetaia* and *Fordilla* ^22^. In specimens studied herein, the lamellar shell layer that is formed from third-order laths may be foliated calcite^26, 27^ or foliated aragonite^28^ (see Supplementary Figs S3 and S4). They may be distinguished via their differing crystallographies, as the main surfaces of the foliated calcitic laths are {1018}^27^ but change to {001} in the foliated aragonite of monoplacophorans^29^. In addition to the different crystallographic orientations, they also show some differences in morphology. The occurrence of many groups of elongated laths with straight or rounded fronts, not toothed patterns, was proposed as foliated aragonite, as in most cases of *P*. *madianensis*.

Crossed foliated lamellar microstructure (CFL) is a new terminology to describe this unique type of shell microstructure. Determination and description are mainly based on features of its exterior surface as vertical section is inaccessible in fossil material. The microstructure studied herein is strikingly similar to foliated aragonite microstructure as both exhibit complex hierarchical growth patterns, but CFL shows a crucial difference in the growth directions of crystals. The alternative sequences of the first-order lamellae in CFL are analogous to that in the crossed lamellar (CL) and the crossed foliated (CF) microstructures of modern molluscan shells. Third-order laths of CFL are generally developed parallel to the growth surface; whereas in CL, they dip at a very high angle (see Supplementary Fig. S5). CFL and CF may be distinguished clearly via their mineralogy, as CFL is composed of aragonite, but CF is composed of calcite. Therefore, CFL exhibits straight or rounded fronts compared with arrow or tooth-like pattern in CF^14^.

Some of the Cambrian molluscan shells appear to have isolated tablets in their internal lamellar layer. Tablets with a rhomboidal outline are common in calcite semi-nacre^30, 31^, while hexagonal tablets are usually observed in some foliated aragonitic shell layers^22, 32^. These textures are straightforward to distinguish based on their differences in morphology and interfacial angle. The interfacial angle of hexagonal tablets is 120°, while angles of 78° and 102° occur in rhomboidal crystals^11^. The isolated tablet is possibly an initial developmental stage during the evolution from foliated aragonite to nacre based on their similarity in hexagonal outline of crystallites^22, 33^.

Examination of shell microstructures in Cambrian mollusks shows the evolutionary trend of loosely-organized lamello-fibrillae in early molluscan shells, through to the well-organized lath-like lamellar types. The lamello-fibrillae is the most widespread of mollusc shell microstructures in the early Cambrian (Terreneuvian) around the world. Commonly, crystals in early molluscan shells are loosely integrated, indicating weak control over biomineralization by organic matrices^10^.

By early Cambrian Stage 3, most mollusks were able to construct their shells with a variety of complex microarchitectures, *e*.*g*. foliated aragonite and isolated tablets^11^. The lath, blade or rod-shaped crystallites of aragonite are likely to have been derived from needle-like fibers via an intermediate stage of acicular laths as both microstructures grew in a similar lateral direction. Such laths may have eventually evolved into hexagonal tablets controlled by organic matrix. It is clear that there was a gradual transition from lamello-fibrillae to fibrous foliated microstructure in the shells of early Cambrian mollusks. Subsequently, this fibrous foliated microstructure was likely to have evolved into foliated aragonite and crossed foliated lamellar microstructure through the control of the arrangement and orientation of the third-order laths into different aggregations. This is seen in the same radial orientations of foliated aragonite, or in the comarginal but opposite orientations of crossed foliated lamellar microstructures (see Supplementary Fig. S6).

Another significant event in molluscan evolution during the early Cambrian is the biomineralization of calcite in the construction of the shell. Recently, lamellae of calcite were reported in *Pelagiella* from early Cambrian Parara Limestone of South Australia^11^. However, our work reveals that the shell of *P*. *madianensis* was exclusively made of aragonite. The origin of calcite in aragonitic shells is more difficult to decipher, but there is a clear trend marked by the transition from widespread, aragonitic mollusk shells during the early Cambrian, to evolution of calcitic shells that became common in the mid-Cambrian^33^. Quantitative analysis of biomaterials during the Cambrian explosion on taxa of high taxonomic rank shows a similar trend. This transition is likely to be dictated by ocean environment, in particular, seawater chemistry (Mg/Ca mole ratio), which played a major role in the initial acquisition of biomineralized skeletons^34, 35^. Seawater chemistry varied through the Ediacaran to Cambrian. The high *m*Mg/Ca ratio is associated with aragonite biomineralization from Ediacaran until the Cambrian Stage 3, and the low *m*Mg/Ca ratio with calcite biomineralization thereafter^36^. Mollusk data coincides well with the oscillation of seawater chemistry, suggesting that environmental changes had major influence on the evolution of biomineralization (see Supplementary Fig. S7).

The late Cambrian has a poor fossil record of mollusks. It was not until the Ordovician when strong nacreous microstructure originated in several molluscan lineages independently, *i*.*e*. bivalves, cephalopods and gastropods^33, 37^. By this time the main types of mineralogies and microstructures observed in modern mollusk shells had evolved. The derived crossed lamellar microstructure and nacre (mother of pearl) steadily became the two most widespread microstructures in shells of bivalves and gastropods due to their unique mechanical properties^38, 39^.

## Methods

The Xinji Formation, yielding abundant Small Shelly Fossils (SSFs), deposited along the southwestern margin of North China block. It rests disconformably on the upper Ediacaran Dongpo Shale and is conformably overlain by the massive dolostone of the Zhushadong Formation. The specimens of *Pelagiella madianensis* from the Xinji Formation are mostly collected from the Chaijiawa section, Longxian County, Shaanxi, North China, approximately 0.5 km northeast to Chaijiawa village.

Rock samples were treated with buffered 10% acetic acid to retrieve acid-resistant microfossils. More than 2000 specimens of *Pelagiella madianensis* were recovered from the acid-resistant residues. Selected specimens were mounted, sputter-coated with gold and examined with a FEI Quanta 400 FEG scanning electron microscope at Northwest University. Approximately half of these specimens are preserved with shell microstructures. Microfossils described below are deposited at the Department of Geology, Northwest University, Xian, China.

## Electronic supplementary material

Cambrian Molluscan Biomineralization
