# Supplementary material for: Complex hierarchical microstructures of Cambrian mollusk Pelagiella: insight into early biomineralization and evolution
Source: Sci Rep. 2017 May 16;7:1935. doi: 10.1038/s41598-017-02235-9 (PMC5434049; doi:10.1038/s41598-017-02235-9)

**Complex hierarchical microstructures of Cambrian mollusk *Pelagiella*: insight into early biomineralization and evolution**

Luoyang Li [1], Xingliang Zhang [1 *], Hao Yun [1], Guoxiang Li [2]

[1] Shaanxi Key Laboratory of Early Life and Environments, State Key Laboratory of Continental Dynamics, and Department of Geology, Northwest University, Xi'an 710069, PR China.

[2] SKLPS, Nanjing Institute of Geology and Palaeontology, Chinese Academy of Sciences, Nanjing 210008, PR China.

[*] Corresponding author at: Department of Geology, Northwest University, Xi'an 710069, PR China. Tel.: +8602988303200; Fax: +8602988363659; E-mail address: xzhang69@nwu.edu.cn

**Supplementary Fig. S1. Preservational bias of *Pelagiella* specimens worldwide.** **(**a). LC063514, specimens collected from the middle part of the Xinji Formation, North China. (b). Amplification of (a), shows the delicate foliated aragonite microstructure. (c). Ajax-1-024, specimens collected from the *Abadiella huoi* trilobite zone in the AJX-M section, Mount Scott Range, South Australia. (d)(e) Amplified images, show the polygonal textures on outer surfaces of internal moulds. (f–i), Specimens were provided by Christian B. Skovsted (Swedish Museum of Natural History). (f). NEG-C.H.O-N-001, collected from Bastion Formation, North-East Greenland, GGU sample 314933. (g). W-NFL-1-001, Forteau Formation, Newfoundland, sample JSP1982-01. (i). Pen-USA-002, Basal Kinzers Formation, Emigsville Member, Pennsylvania. (e)(h). Amplification of (g) and (i), shows the

internal moulds and coarse crystals. Scale bars are (a)(c)(i) 100µm; (b) 10µm; (d) 50µm; (e) 1µm; (f) 300µm; (g) 200µm; (h)(j) 5µm.

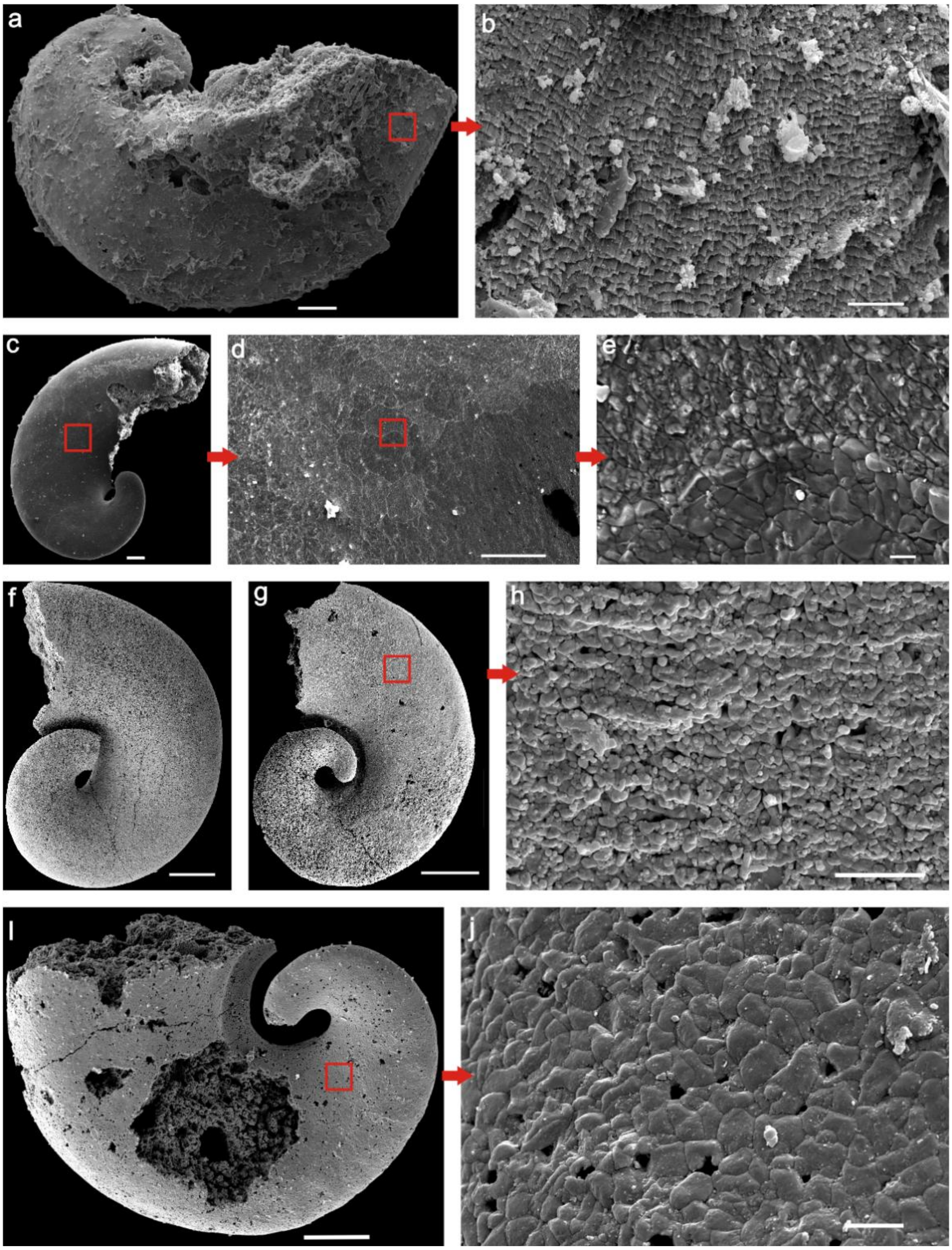

**Supplementary Fig. S2. Nanogranules in nacre of living gastropods.** Specimens were purchased online, which were collected from the Bohai Sea, Western Pacific. (a). LG001_013, oblique fracture of the shell shows the nacre (mother of pearl). (b). RLG001_012, interior surface shows the nacrous tablets with the central hole. (c). RLG001_005, acid-etched nacre shows the aragonitic nanogranules. Scale bars are (a) 10μm; (b)(c) 1μm.

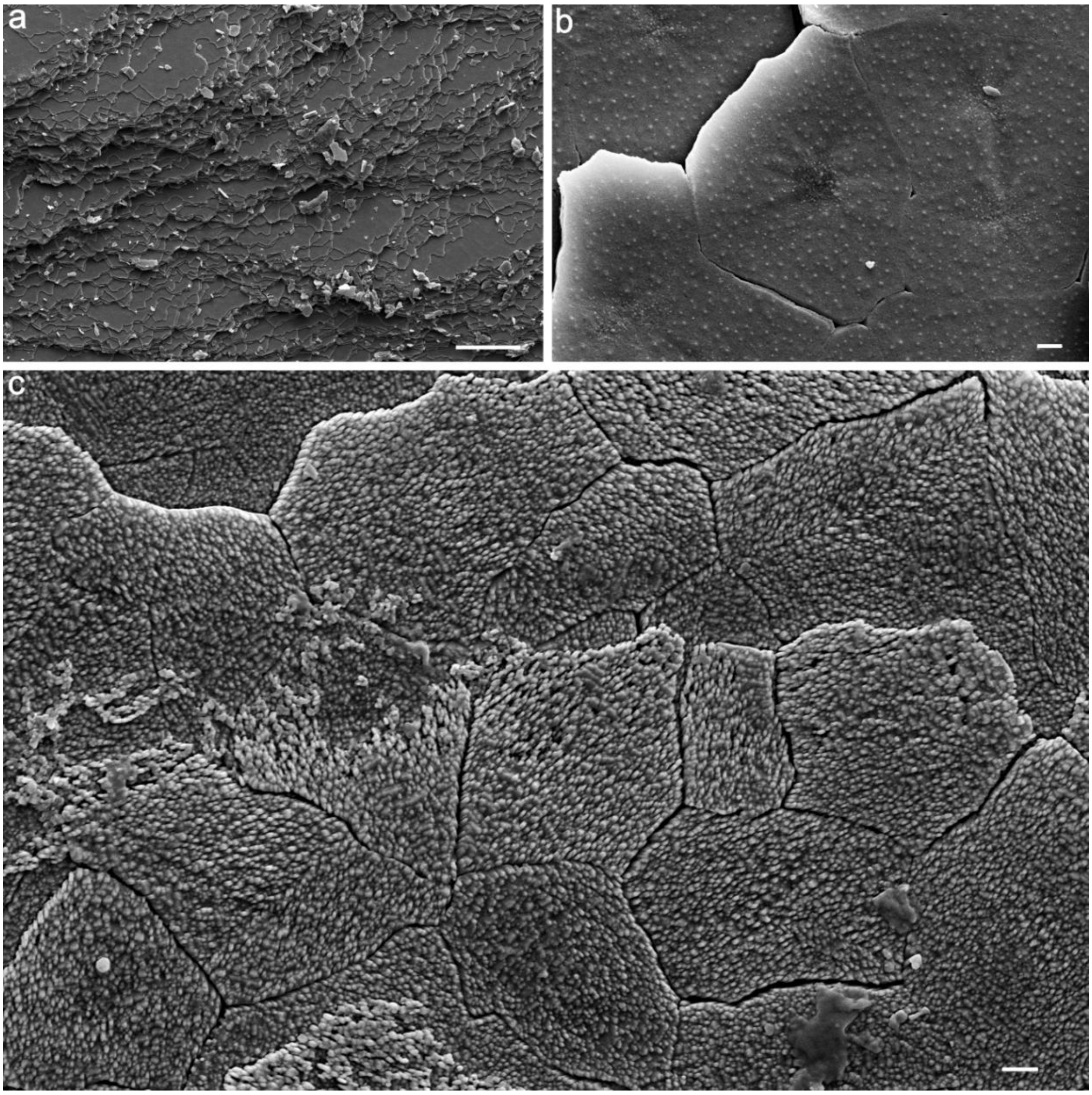

**Supplementary Fig. S3. Foliated calcite microstructure of living bivalve oyster.** Specimens were purchased online, which were collected from the Bohai Sea, Western Pacific**.** (a). O1-1-018, interior surface, foliated calcite align in various orientations. (b). O1-1-019, interior surface, laths of foliated calcite have arrow-shaped fronts. (c). O3-2-004, interior surface. (d). O1-2-002, exterior surface, (e). Amplification of (d), shows tooth-like pattern of calcitic crystallites. Scales bars are: (a) 50μm; (b) 20μm; (c) 5μm; (d) 100μm; (e) 10μm.

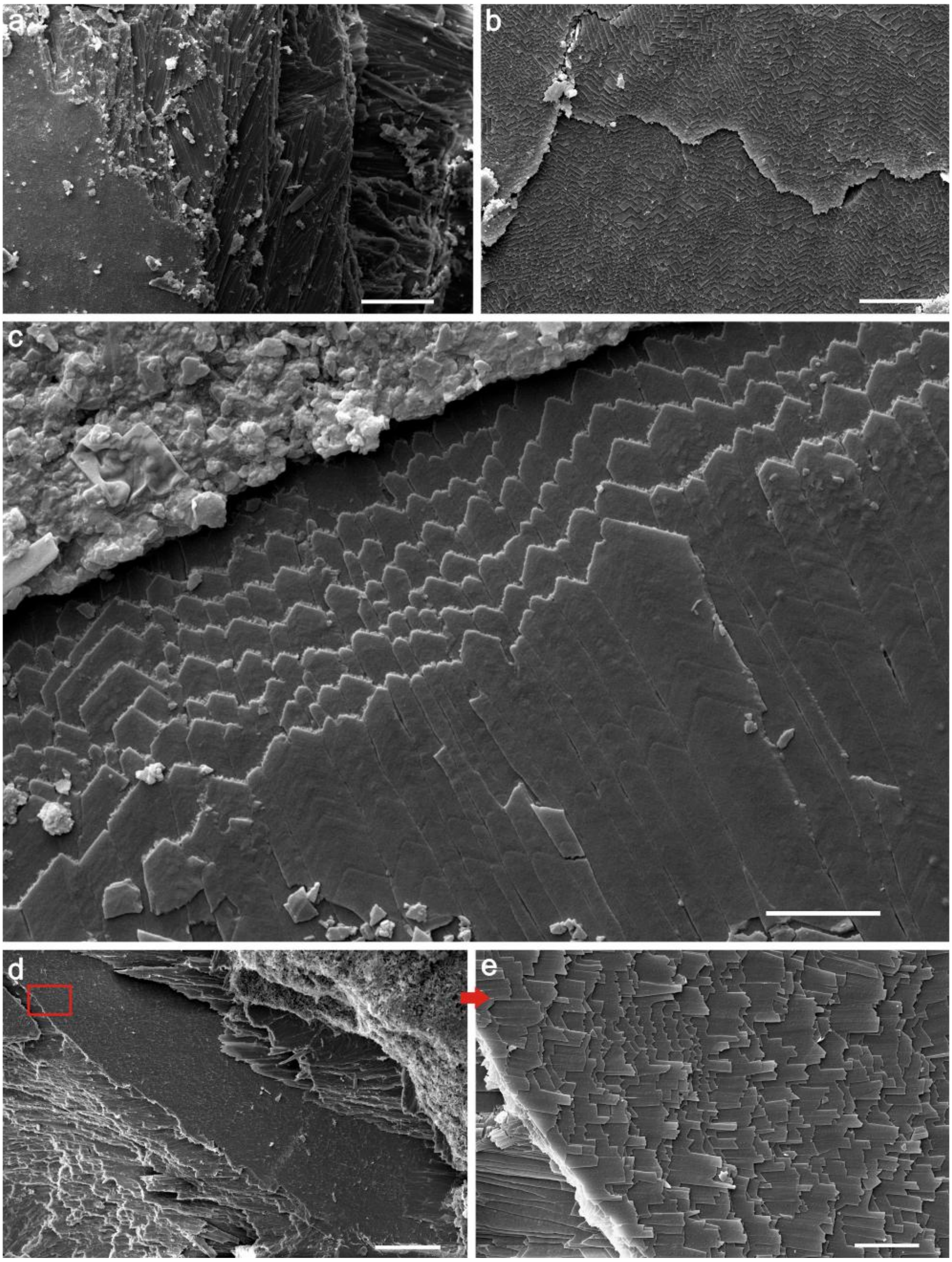

**Supplementary Fig. S4. Foliated aragonite microstructure of Cambrian bivalve *Pojetaia runnegari*.** Specimens of *P. runnegari* co-occur with *P. madianensis* in the same bed of Xinji Formation. (a, b) The subunit of lath-like crystals arranged in clockwise direction with straight front perpendicular to shell edge. (c, d) The second-order folia with a straight front and a regular stepwise pattern. (e) New crystals seem to be inserted between adjacent parent folia in the same orientation. Scale bars are (a) 200µm; (b)(c)(e) 10µm; (d) 50µm.

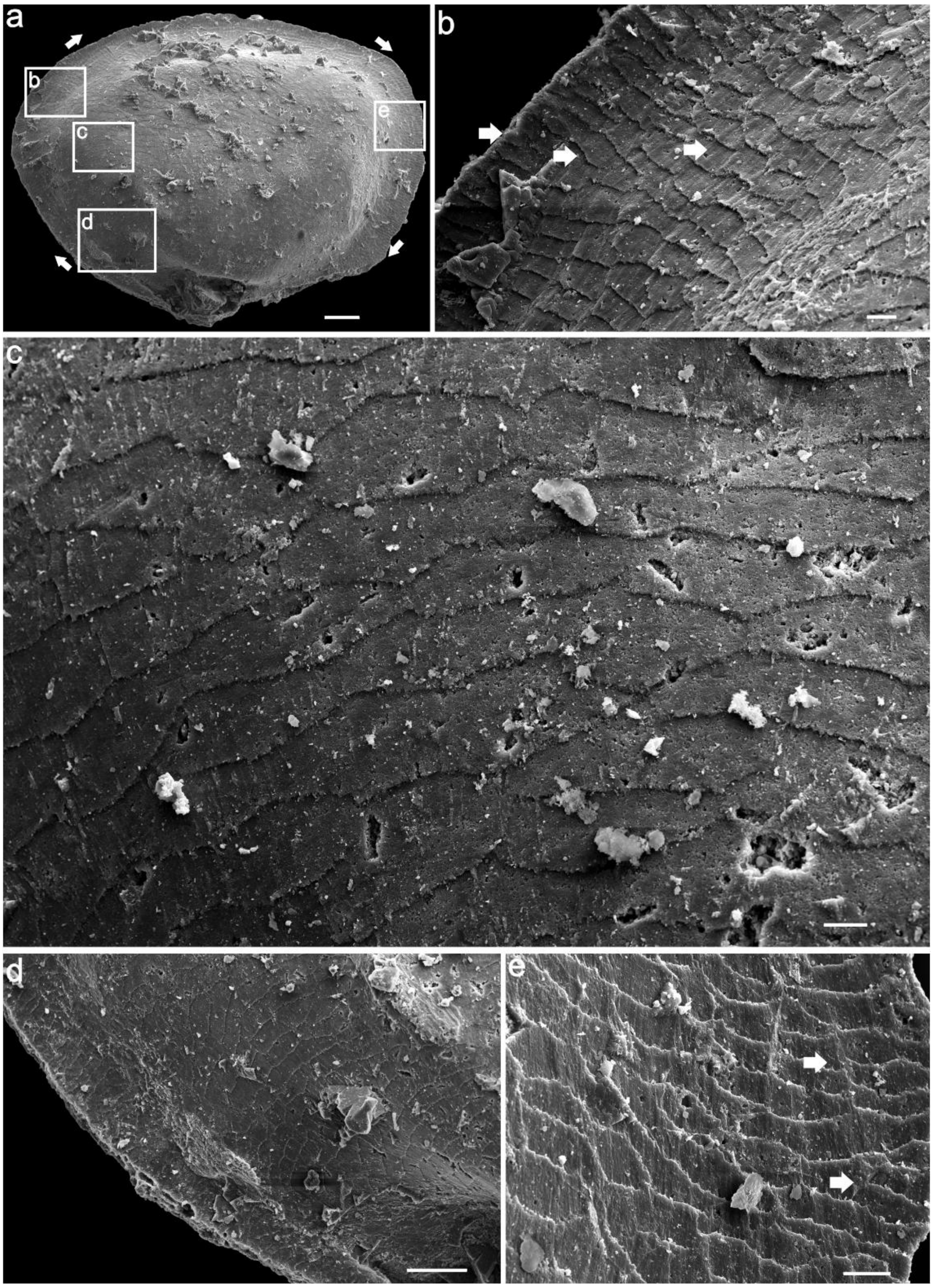

**Supplementary Fig. S5. Crossed lamellar microstructure of living gastropod.** Specimens are purchased online which were collected from the Bohai Sea. (a) Vertical section, shows two layers of crossed lamellar microstructure (CL) arranged in radial and comarginal directions respectively. (b) Horizontal section, shows the first-order lamellae of inner layer arranged in a comarginal direction and perpendicular to outer shell surface (c) Amplification of (b), shows the third-order lamellae. (d) Fractures show two layers with CL. (e) Interior surface of the shell. (f) Amplification of (e), third-order lamellae between adjacent first-order lamellae are arranged in a comarginal but opposite direction. Scale bars are (a) 100μm; (b)(e) 50μm; (c) 1μm; (d) 500μm; (f) 5μm.

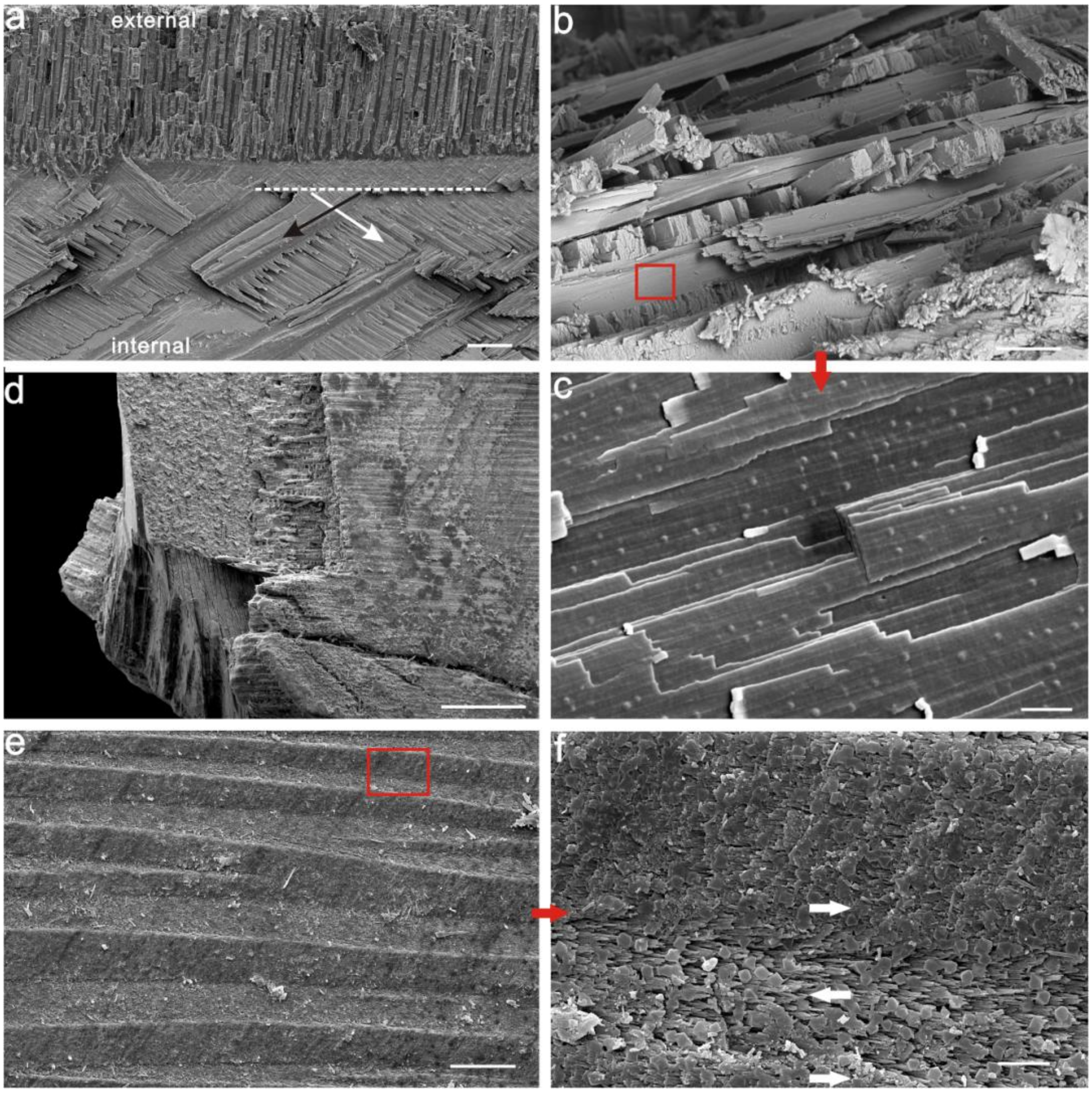

**Supplementary Fig. S6. Microstructural evolution of molluscan shells during Cambrian.** The Neoproterozoic ancestor of mollusks is hypothesized to possess an unmineralized soft body. Hard shells and their microstructures become diverse during the Cambrian. Molluscan shells in the early Cambrian, Terreneuvian are mainly composed of lamello-fibrillar or fibrous microstructure, which consists of needle-like crystallites of aragonite. Later, fibers evolve into higher ordered units, *e.g.* acicular laths, laths, folia and tablets, and hence well-organized microstructures like fibrous aragonite, foliated aragonite and hexagonal tablet evolved. By the time of Cambrian Age 3 rhombic calcite appears in the fossil record, and foliated calcite and semi-nacre calcite microstructures evolved.

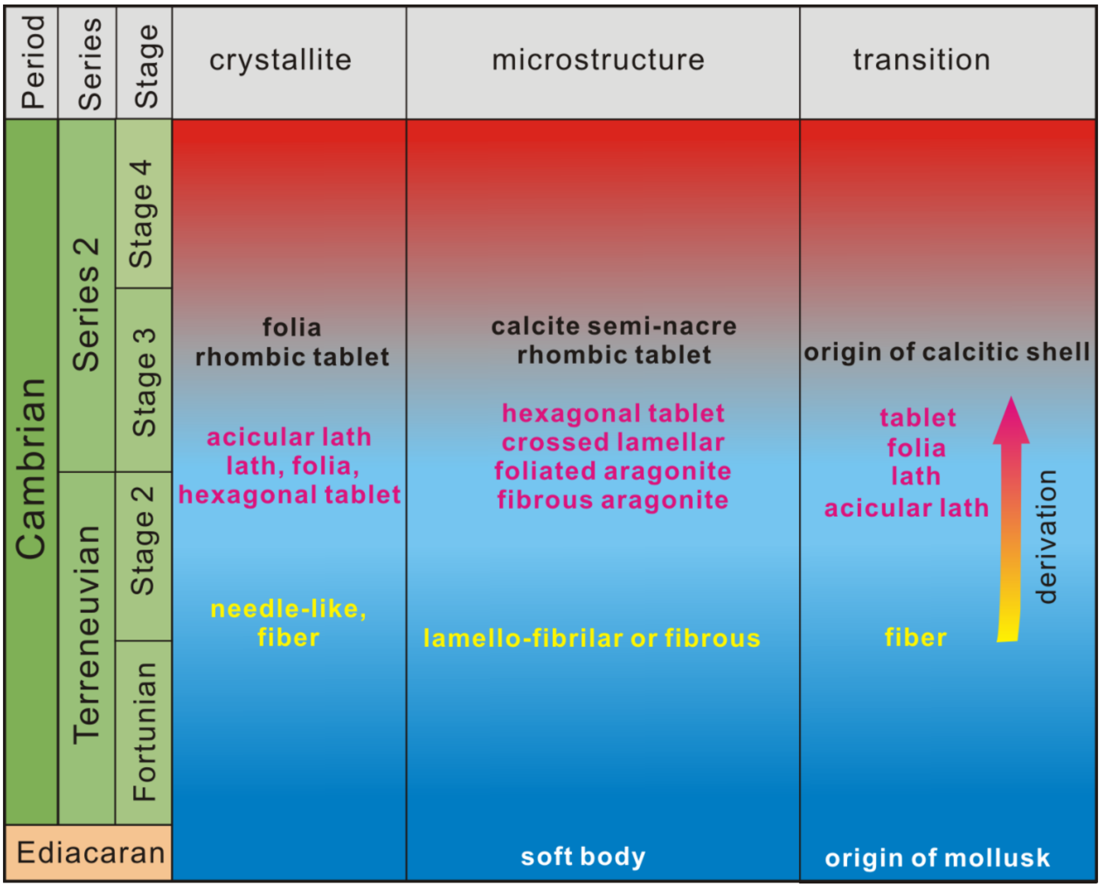

**Supplementary Fig. S7. Mineralogical evolution of Cambrian molluscan shells on genus level.** First appearance of molluscan shell on genus level (31 genera) with certain mineralogy from upper Ediacaran to Cambrian Series 3. The data of molluscan shells are collected from previous work by other authors: Runnegar 1985, Runnegar & Jell 1976, Bengtson et al. 1990, Gravestock et al 2001, Kouchinsky 1999, 2000, Feng & Sun 2003, Ushatinskaya 2005, Parkhaev 2006, Vendrasco and Checa 2015, Vendrasco et al 2010a, b, 2011, 2015 and fluid inclusion data are from Petrychenko et al. (2005) and Porter (2010). Biominerals are exclusively aragonite during Cambrian Terreneuvian, while about 70% of them during Cambrian Series 2 and 3 are composed of calcite.

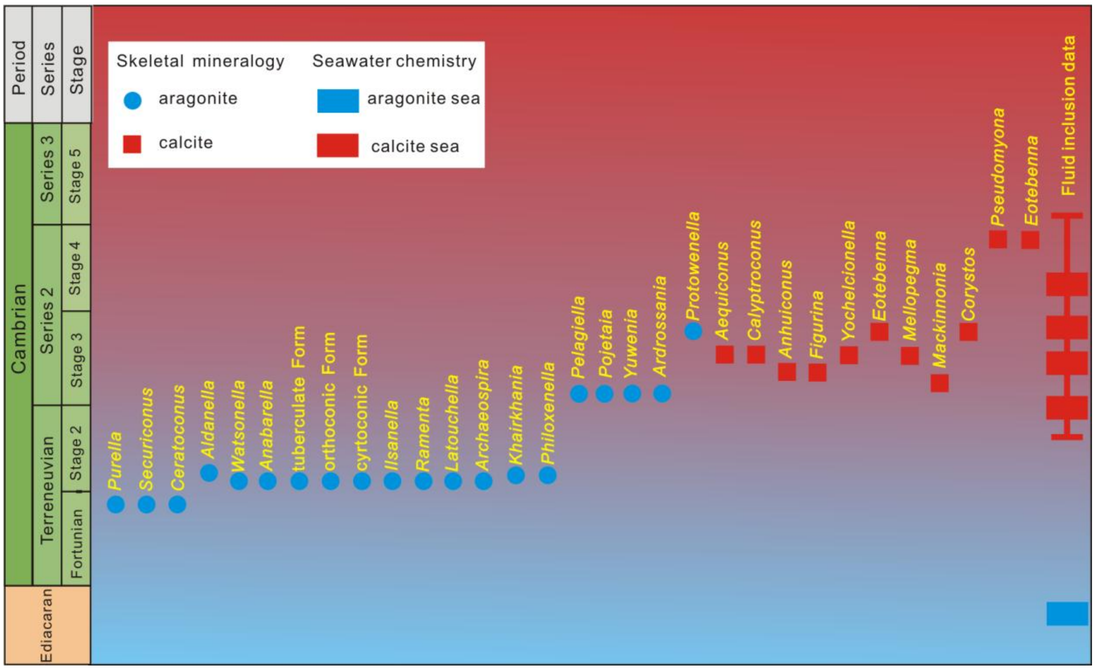

Supplement: Supplementary file 1 — Cambrian Molluscan Biomineralization [file 41598_2017_2235_MOESM1_ESM.pdf]
